# Implementation of a Pediatric Early Warning Score to Improve Communication and Nursing Empowerment in a Rural District Hospital in Rwanda

**DOI:** 10.9745/GHSP-D-20-00075

**Published:** 2020-12-23

**Authors:** Shela Sridhar, Alexis Schmid, Francois Biziyaremye, Samantha Hodge, Ngamika Patient, Kim Wilson

**Affiliations:** a Boston Children’s Hospital, Boston, MA, USA.; b Inshuti Mu Buzima-Partners in Health Rwanda, Kigali, Rwanda.; c Kirehe District Hospital, Kirehe, Rwanda.

## Abstract

**Background:**

Pediatric early warning (PEW) scores represent a “track-and-trigger system” that identifies clinical deterioration in a patient’s condition in the hours preceding a sentinel event. Before implementation, nurses reported feeling unprepared to identify and advocate for acutely ill patients owing to a lack of skills, vocabulary, and agency. We implemented a Pediatric Early Warning Score for Resource-Limited Settings (PEWS-RL) with nurses in a rural district hospital in Rwanda. Although PEW scores can improve clinical outcomes, empowering nurses in resource-limited settings to discuss patient acuity with physicians is a critical first step. Our primary aims were to train nurses to obtain more accurate vital signs and assess their importance as early warning signs of clinical deterioration and use PEW scores to improve communication between nurses and physicians.

**Implementation:**

The PEWS-RL tool implementation began with a training program that was created through discussions with nurses, physicians, and the medical director of the hospital. The program included lectures and application of learned skills through direct clinical mentorship of nurses, as well as training of physicians regarding PEWS-RL as a communication tool.

**Evaluation:**

The PEWS-RL protocol was evaluated based on pre- and post-tests to assess improvement in nurses’ knowledge and skill, as well as skills assessments of accurate recognition of clinical deterioration. All 6 nurses passed skill testing with >80% accuracy. Nurses’ feelings of empowerment to advocate for patients and to escalate care were assessed through pre- and post-training interviews. Nurses described increased confidence in calling for physician support.

**Discussion:**

Implementation of PEW scores increased nurses’ technical skills and feelings of confidence and empowerment; however, the low-resource setting presented major challenges. Barriers to sustainable implementation include the rapid ward staff turnover as well as limited physician buy-in. Nevertheless, the PEWS-RL tool has the potential to empower nurses and improve patient outcomes if fully embraced by staff.

## Abstract (precis)

Implementation of the Pediatric Early Warning Score for Resource-Limited Settings tool improved nurses’ competency and confidence in their triage capabilities. This tool has the potential to improve patient outcomes. However, staff turnover and limited physician buy-in were barriers to sustainability of the tool in low-resource settings.

## Teaser

**Key Findings:**

- Nursing staff felt more empowered to communicate clinical findings to the physician team following the Pediatric Early Warning Score for Resource-Limited Settings tool training and implementation process.
- The process of implementing the tool triggered more calls from nursing staff to the physician teams to initiate early intervention.

**Key Implications:**

- The Pediatric Early Warning Score for Resource-Limited Settings tool has the potential to improve competency and confidence of nurses in their triage capabilities. Although traditionally implemented in tertiary care centers, program managers should consider implementing this tool at the district hospital (secondary) level as well.
- Physicians and nurses both play crucial roles in triaging systems. Therefore, program managers should consider engaging both groups with the tool before and during implementation.

## BACKGROUND

### Responding to Increased Child Mortality Rate

Kirehe District Hospital (KDH) is a public hospital in rural Rwanda, supported by a partnership with the nongovernmental organization (NGO) Inshuti Mu Buzima, a local organization of Partners in Health. KDH serves a catchment area of approximately 340,000 people; or 13% of Rwanda’s population, including a large refugee settlement with 57,000 residents.^1^ About 50% of the population in Kirehe is under the age of 17. KDH has a busy general pediatric ward, with 60–120 pediatric admissions per month of children aged 1 month to 15 years old. The staffing model for the pediatric ward includes 1 or 2 nurses caring for 10 to 30 pediatric patients, supported by a general physician covering the pediatric ward as well as a 40-bed neonatal ward. The total medical staff comprised 6 pediatric nurses and 10 physicians. In addition, a U.S.-trained pediatrician affiliated with Partners in Health works with the hospital to conduct on-site clinical capacity building for several months each year.

In 2018, hospital staff noted rising mortality rates in the pediatric ward. A chart review between May and October 2018 indicated that for some months the child mortality rate was as high as 6% and the average for the 6 months about 3%. The majority of deaths were associated with sepsis or pneumonia resulting in respiratory failure, often as a consequence of inadequate recognition of altered mental status and respiratory fatigue. Death occurred an average of 7 days after admission, with a minimum of <24 hours and maximum of 27 days after admission. Potential etiologies for death after 7 days of admission may have been iatrogenic (management of patients), but we suspected there was also poor recognition of warning symptoms and clinical progression of disease that may have been missed in the days leading to mortality later in the hospital course.

A variety of factors can contribute to clinical deterioration or high rates of mortality in children with sepsis or pneumonia. However, the incidence of such events can be significantly lower in similar settings where early recognition of deterioration and prompt initiation of treatment or early transfer to higher levels of care have been initiated.^2^

More than 95% of pneumonia-related deaths occur in low- and middle-income countries. However, little data exists on quality-of-care indicators and practices around pneumonia care in these contexts.^3^ This paucity of data suggests a gap in assessment of quality of care in pediatric populations.

### Understanding Hospital Care Delivery Factors

To better understand hospital-based care delivery factors that could be contributing to high pediatric mortality, we conducted key informant interviews with the 6 pediatric nurses and 4 physicians. After observing multiple deaths on the pediatric ward, the Partners in Health-affiliated pediatrician hypothesized that a driver of the mortality rate was inadequate communication between nurses and physicians. This was observed at 2 separate Partners in Health supported hospitals. However, because KDH was the busier facility at the time, it was chosen for the intervention.

We conducted informal interviews with 4 physicians to gain a background understanding of physicians’ perceptions regarding nursing competencies. The 4 physicians interviewed were those who spent the greatest amount of time on the pediatric ward. We asked the 6 pediatric nurses questions regarding their comfort level with triaging and communication processes. The interviews also included open-ended questions to draw out additional themes. We asked the interview questions in English and used an interpreter who spoke Kinyarwanda and English to translate. Interviews with nurses and physicians lasted no more than 30 minutes. Common themes that emerged for both nurses and physicians included constraints on time and human resources, which compromised the clinicians’ ability to appropriately prioritize patients and complete tasks. Nurses also cited gaps in knowledge and skills in identifying and subsequently reporting the status of critically ill patients as creating a barrier to timely care. Nursing leadership highlighted that nurses felt disempowered to advocate for deteriorating patients. They reported lacking a common language around assessment of critical illness with physicians and therefore feeling unprepared to highlight the acuity of a patient’s condition and effectively advocate for them. Other nurses reported that their concerns were sometimes dismissed by physicians who would respond by saying:

*Pediatric vitals are different.*

General physicians cited concerns with the accuracy of vital signs reported by nurses as a key barrier in assessing the severity of pediatric illness. Multiple physicians indicated that:

*If I want to believe the vitals, I take them myself.*

### Using Pediatric Early Warning Score for Triage

Although time constraints and human resource allocation are subject to financial constraints, clinical processes such as accurate triage and communication are modifiable factors with minimal financial burdens on low-resource hospital systems. Pediatric early warning (PEW) scores represent a triage “track-and-trigger system” that can accurately identify up to 85% of children who will experience clinical deterioration, such as cardiac arrest or severe respiratory compromise, sometimes as early as 11 hours before the sentinel event.^4^ The scores are a mechanism that can be used to modify triage systems and standardize communication regarding acutely ill children. PEW scores have primarily been used in high-income countries, but they were recently adapted for use in resource-limited settings (Pediatric Early Warning Score in Resource-Limited Settings [PEWS-RL]) and validated in a tertiary care setting in Rwanda.^5^ The PEWS-RL uses basic clinical assessments including respiratory rate, respiratory distress, heart rate, temperature, blood pressure, oxygen use, and mental status. It demonstrated a 92% sensitivity and an 87% specificity in identifying children at risk of clinical deterioration.^6^

Pediatric early warning scores represent a triage “track-and-trigger system” that can accurately identify up to 85% of children who will experience clinical deterioration.

In interviews, nurses cited that a barrier to timely care were gaps in knowledge and skills in identifying and reporting the status of critically ill patients.

To improve early recognition and communication of clinical deterioration in pediatric patients by nursing staff, we aimed to implement a standardized triage system including a standardized clinical assessment for patients at risk for clinical deterioration in our inpatient pediatric ward. We hypothesized that this intervention would improve nurses’ ability to accurately identify critically ill patients, improve communication about critical patients by creating a common language with physicians, and prompt a timely physician response to evaluate and initiate the appropriate medical management for a child whose condition is deteriorating. In this report, we describe our process for implementation of the PEWS-RL at the district hospital level, including areas of success, challenges, and lessons learned.

## IMPLEMENTING PEW SCORES

We reviewed several versions of the PEW triage tools collaboratively with staff and leadership at KDH, including the medical director, clinical director, and the primary general practitioner who rounded on the pediatric wards. PEW systems include 2 components: a score calculated using vital signs at prescribed intervals during a child’s hospitalization and a response system, which may be as simple as contacting a physician, that is activated if a specific score threshold on the tool has been reached.^7^ ^,^ ^8^ Early warning scores commonly evaluate and score vital signs as well as clinical exam assessments, such as level of consciousness, capillary refill, or work of breathing.^8^ No general consensus exists regarding which components are essential, the frequency with which they should be recorded,^5^ or the thresholds and scoring mechanism that indicate clinical concern.^5^ ^,^ ^8^ Few versions have been evaluated in resource-limited settings^5^ ^,^ ^7^ where staffing ratios and the level of nurses’ training differ.^5^ After an analysis of PEW tools and initial conversations with staff, we determined that it would be best to focus on objective data (basic vital signs alone) without including clinical assessment. Given that the PEWS-RL met these criteria and had previously been validated in Rwanda in a tertiary hospital setting, we decided to implement the same version in our hospital for consistency and potential nationwide scalability in the future.

PEW systems include 2 components: a score based on a child’s vital signs and a response system that is activated if a specific score is reached.

The PEWS-RL tool (Figure) is purposefully composed solely of vital signs that are attainable with minimal equipment or assessment ability. This approach was taken because clinical assessments, such as blood pressure and respiratory effort, are often not examined due to a lack of trained personnel and availability of pediatric-sized equipment.^5^ This tool was utilized across the pediatric age range (1 month to 15 years). Our PEW score included respiratory rate, heart rate, temperature, and mental status; each was scored at 1 point. Physician notification was triggered at a score of 3 on admission or an increase of 3 points on subsequent assessments. Blood pressure was initially included in the assessment of the PEWS-RL; however, based on discussions with the research team at the University Teaching Hospital of Kigali, the sensitivity and specificity of the tool did not notably change when blood pressure was removed. Therefore, we did not include it in our score.

**FIGURE:**
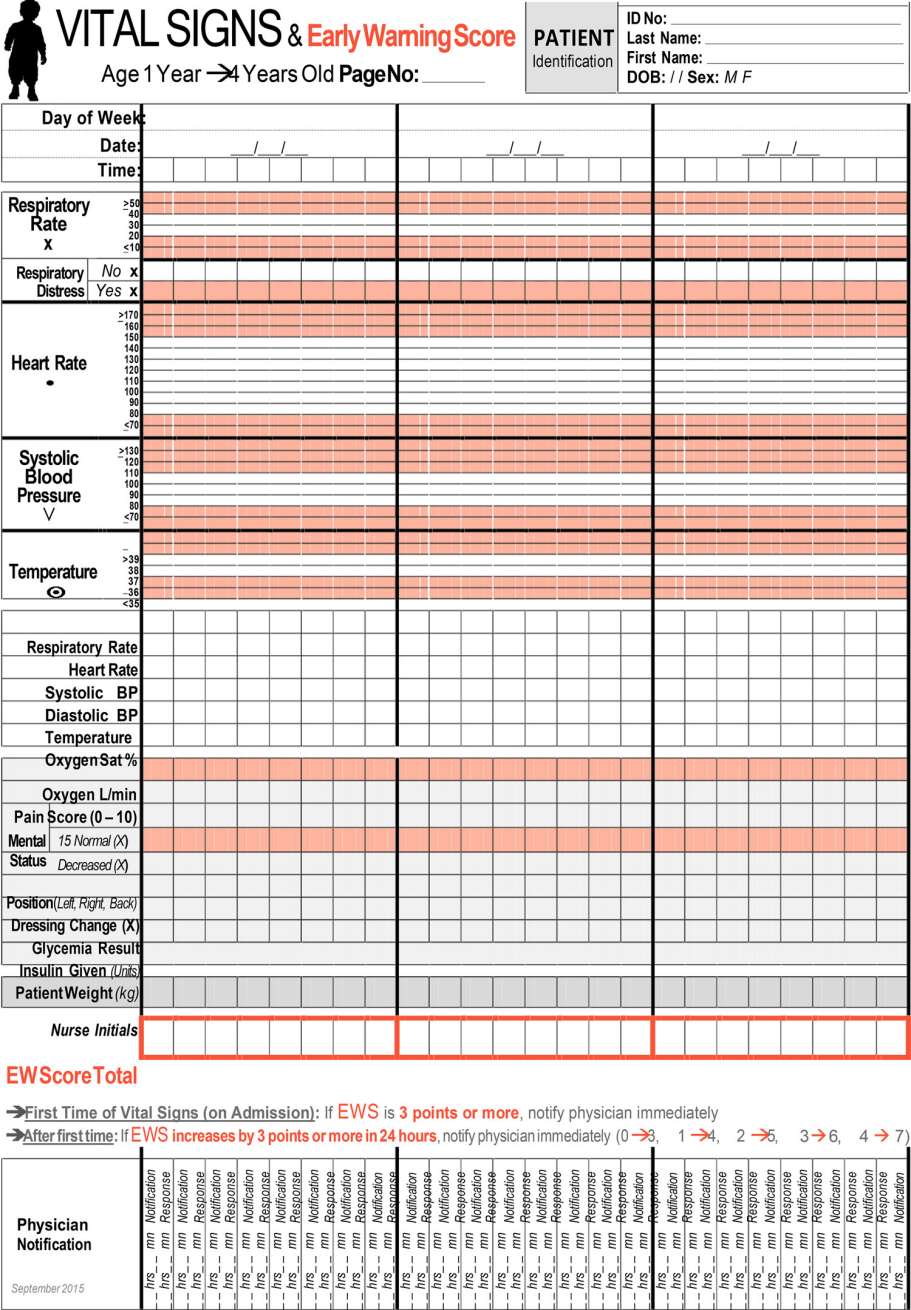
Pediatric Early Warning Score for Resource-Limited Settings Tool Used at the University Teaching Hospital of Kigali, Rwanda, and Kirehe District Hospital Abbreviation: BP, blood pressure.

### Training Approach

The initial implementation of PEW scores started with a training program for 6 nurses and 10 physicians over a 2-week period in November 2018. A visiting U.S.-based pediatric nurse specialist provided 1–2 hours of on-site didactic training per day to the pediatric ward nursing staff, focusing on the clinical importance and implications of abnormal vital sign values. Although many (but not all) physicians and nurses are exposed to emergency triage and assessment training in school, ongoing mentorship or recertification is uncommon. The remainder of the day focused on application of learned skills and direct clinical mentorship through “real-time” patient assessments and active feedback at the bedside. Nurse training included bedside mentoring during morning rounds and didactic sessions each afternoon with continued bedside teaching through the day. The training was incorporated into the nursing work flow to the extent possible to be minimally disruptive in an already understaffed environment.

Along with clinical and didactic training, nurses were provided stethoscopes so they could manually check heart rates and blood pressures as a secondary validation of the cardiorespiratory monitor used on the unit. They were also provided individual pulse oximeters to be used as a secondary check on the existing monitor. Equipment was distributed at the start of the training and used throughout the 2-week course.

General physicians were given 2 lectures dedicated to understanding the PEW score and response system by the NGO pediatrician. Lectures for nurses focused on assessment and reporting of the score, and lectures for physicians focused on responses to different scores and critical thinking around common case scenarios. In addition, the pediatrician rounded with the clinical team each morning for the 3 months following the training program and provided ongoing low-dose, high-frequency mentorship for both rounding physicians and nurses in using and interpreting the PEWS-RL. PEW scores for patients were reviewed each morning to assess for completion and to facilitate discussion of any challenges encountered during the implementation. Through this process, PEW score documentation was integrated into ward rounds and the existing work flow for both nurses and physicians.

### Assessment of Outcomes

#### Nursing and Physician Knowledge, Skills, and Clinical Practices

We conducted an evaluation of the impact of PEWS-RL implementation at KDH on nursing and physician knowledge, skills, and clinical practices using interview data and process measures. Our measures included changes in nursing knowledge and skill in accurate recognition of clinical deterioration, changes in nursing physician communication before sentinel events, and nursing confidence levels in the communication of clinical findings to physician staff. The PEWS-RL triage system includes both the risk score obtained by the nursing staff and the responsiveness of the physician team. Our assessments focused on the primary objective of nursing competency and communication rather than the response component of triage systems.

Our evaluation of the impact of PEWS-RL implementation on nursing and physician knowledge, skills, and clinical practices was based on interview data and process measures.

Nursing knowledge and skills in accurate recognition of clinical deterioration was evaluated using pre- and post-training written tests and clinical skills assessments. Skill competency of KDH pediatric nursing staff was evaluated by the pediatric nurse specialist using a standardized checklist immediately after the 2-week training program. The objective exam focused on their ability to obtain manual vital signs and calculate a PEW score. The clinical competency form used during the assessment is outlined in the Supplement. A numeric score as well as written feedback was provided. All 6 pediatric nurses independently passed skill testing with >80% accuracy. In addition to the clinical skills assessment, a written exam was given on the first and last day of training to assess the clinical knowledge necessary to adequately utilize the PEWS-RL as intended for screening and response activation. The average pretest score for nurses was 66% with a range of 53%–80%. The average post-test score was 81% with a range of 67%–100%.

#### Nursing Communication with Physicians

We conducted a qualitative assessment of the impact of our training on nursing communication with physicians, focusing on the nurses’ level of empowerment in patient advocacy and escalation of care around sentinel events. Pre- and post-training structured interviews of the nursing staff were conducted by an NGO nurse mentor individually and confidentially and in their primary language to promote more open communication. In addition, nurses completed a written survey of their communication practices and comfort level in escalating care before and after completing the 2-week training program. The survey included Likert-scale questions with declarative statements such as “I feel comfortable asking physicians questions” as well as free-form answers to questions around communication such as “How do you feel communication between nurses and physicians affects patient care?” Before training, 2 of 6 nurses felt as though their clinical assessments were often dismissed by physicians. Post-training, nurses stated feeling more confident in their ability to advocate for patients. One nurse articulated:

*Now I have a tool to back me up when I call the doctor.*

Nurses described increased confidence calling for physician support:

*[We] have something to say.*

Changes in nursing clinical communication practices were also evaluated by retrospective chart review. In the 6 months before the implementation of our PEWS protocol, only 8 of 30 patients (27%) who had been transferred or died had a recorded call to a physician in the 11 hours preceding the sentinel event (transfer to a tertiary care center, resuscitation by KDH staff, or death). However, in the 2 months immediately following the intervention, we found that the physician was called 63% of the time (7/11 patients) before a sentinel event. In the 6 months before the intervention, there was a 3% mortality rate and a 3% transfer rate for all patients in the hospital. In the 2 months after our intervention, the mortality rate was not significantly different, but the transfer rate had increased by 11%. A chart review also demonstrated an increase in physician documentation outside traditional rounding hours, suggesting an increase in physician response frequency; however, physician response times will need to be evaluated over time in subsequent iterations. Although the data collected from this training program are not adequately powered to make definitive conclusions regarding the effect of PEW scores on clinical outcomes or physician response times, the initial inferences are promising and merit further investigation.

## DISCUSSION

Implementation of our PEW score created an opportunity for vital nursing education on high-quality assessment of vital signs and a deeper clinical understanding of underlying pediatric physiology. The PEWS-RL implementation also empowered nurses, provided them with a model, and mentored them on the tools to communicate their assessments to physicians. The public hospital-NGO partnership provided the opportunity for nurses and physicians to observe communication between an external nurse and physician, which modeled important skills in open multidisciplinary communication and physician trust in nursing assessment skills. Given the immediate increase we saw in recorded calls to physicians being documented and postintervention interviews with nursing staff, the PEWS-RL tool may be effective even on a busy ward with limited staff and resources.

The PEWS-RL implementation empowered nurses, provided them a model, and mentored them on the tools to communicate their assessments to physicians.

### Challenges and Lessons Learned

Although nursing knowledge and skills demonstrably improved and nursing staff reported feeling empowered, we encountered several challenges during the implementation of our protocol. Our most significant challenge was in motivating physician engagement. We provided lectures for physicians over the course of the training, but our interactions and reports from nursing staff suggested that the physicians were less interested than the nursing staff in using the PEWS-RL. The reasons for this finding are likely multifactorial. The primary focus of our efforts in training was toward the nursing staff, and we included only 2 training sessions and no formal mentorship process for physicians who did not round on the pediatric ward during the implementation phase. Additionally, of the 16 physicians on staff at the time of the intervention, only 4 were able to spend time on the pediatric wards during the initial implementation of the protocol. Finally, as our focus was on nursing empowerment, we failed to involve the physicians in the planning process of protocol development and implementation, likely leading to inadequate physician understanding and involvement in the protocol.

The critical lesson learned was the importance of engaging physicians and nurses together. We were able to implement the first steps in a triage system,^8^ that is, nursing recognition and empowerment to communicate clinical findings. However, changes to outcomes and mortality will require true physician engagement and understanding of the PEW score tool, its implications, and how to respond to nursing concerns. As noted in the PEW score literature, to be effective in reducing morbidity and mortality, the tool needs to be implemented within a system that is able to respond to the needs of the child; specifically, a provider or a team that has the ability to not only accurately assess the patient and recognize anomalies, but to also implement appropriate clinical interventions.^7^ ^,^ ^9^ ^,^ ^10^ Our PEWS intervention focused on addressing the first steps of recognition and empowerment. However, we did not address the subsequent step, which requires “the assistance is readily available and appropriately skilled…”^8^ Physician engagement can be further accomplished by ensuring leadership and ownership of the tool implementation by the physicians. Additionally, appointing a physician leader in pediatrics may be useful to create physician buy-in and organizational accountability.

The critical lesson learned was the importance of engaging physicians and nurses together.

Although we attempted to educate physicians and nurses side-by-side during medical rounds, we should have placed a greater focus on individual-physician coaching to address the aspect of skilled assistance in response to recognition of illness. Furthermore, before implementation, we did not adjust the physician schedule to ensure that all medical staff rotated through the ward during the training. This decision was made to minimize disruptions to work flow in the hospital as well as minimize administrative burden on the clinical director who managed the schedules. This reasoning also informed why we did not have additional meetings during the training with the entire staff to review and address problems that were noted, but rather managed them on an ad hoc, daily basis. However, in subsequent programs, the short-term disruption may be acceptable if longer-term clinical benefits can be derived.

Other barriers to sustainable implementation of this protocol included the rapid turnover of staff on the ward and a loss of equipment following the training. The chief of nursing was transferred during our training and 1 week later another member of the nursing staff left and was replaced by a new staffer. Next steps to mitigate this limitation include creating an on-boarding system for pediatric nurses as well as new physician staff. Additionally, to minimize the removal of stethoscopes and oximeters from the ward, they should be tagged with large, bulky labels or affixed to a mobile cart, or the work flow should be changed to include retrieval of locked equipment at the start of each nursing shift. Although we are encouraged by the feedback from our nursing staff, another limitation was our status as an outside organization. Survey answers may have been biased due to cultural tendencies to avoid criticizing the system or a desire to provide positive answers.

### Limitations

In addition to the procedural challenges we experienced, important limitations in the assessment and interpretation of the outcomes should be considered. Our assessment focused on nursing competencies, with minimal evaluation of physician responses. This approach prevented us from measuring clinical outcomes and timeliness of responses, which are critical to any effective triage system. Additionally, the program was conducted at one hospital with a small number of nurses. To achieve statistical significance for nursing competencies, this program would need to be conducted across multiple sites or over multiple iterations at KDH. Finally, although the PEW score has been validated in tertiary care centers, it has not yet been validated in district-level hospitals where fewer interventions are available to inform the medical response.

## SUMMARY AND CONCLUSIONS

Early recognition and response to clinical deterioration are essential to improving outcomes in pediatric care in hospitalized patients in resource-limited settings but can be challenging. Initial barriers can include limitations in nursing knowledge and skills in pediatric triage and lack of nursing empowerment in escalating concerns for timely physician response. Training and implementation of the PEWS-RL resulted in demonstrable improvements of both technical skills and feelings of confidence and empowerment among the nursing staff. Challenges and next steps in quality improvement and implementation remain, including the need to address equipment availability and security and the implementation of approaches to improving physician training and buy-in.

However, it remains to be seen if the subsequent steps of the track-and-trigger system can be improved with increased physician involvement in the implementation process. This next step is crucial given that our triage system would ultimately be incomplete without an appropriate response and intervention system.^8^ ^,^ ^11^ The next steps are to design an adequately powered study across multiple district hospitals to evaluate the feasibility and effect of PEW scores in low-resource rural settings. These studies should start by focusing on delivery of care at the hospital level including physician–nursing communication, response times, and appropriateness of medical management to PEW triggers. Based on our implementation experience, it will be essential to conduct intensive nursing and physician training simultaneously with a dedicated review process. Nevertheless, based on our initial assessment, the implementation of PEW scores in a rural district hospital in sub-Saharan Africa has the potential to empower nurses and improve patient outcomes in low-resource settings if fully embraced by staff.

## Supplementary Material

20-00075-Sridhar-Supplement.pdf

## Notes

**Peer Reviewed**

**First published online:** October 15, 2020
