# Supplementary material for: Implementation of a Pediatric Early Warning Score to Improve Communication and Nursing Empowerment in a Rural District Hospital in Rwanda
Source: Glob Health Sci Pract. 2020 Dec 23;8(4):838–45. doi: 10.9745/GHSP-D-20-00075 (PMC7784060; doi:10.9745/GHSP-D-20-00075)
Supplement: 20-00075-Sridhar-Supplement.pdf [file 20-00075-Sridhar-Supplement.pdf]

Partners In Health | Inshuti Mu Buzima

CLINICAL COMPETENCIES

COMPETENCY STATEMENT: The nurse safely and effectively obtains vital signs on a pediatric patient and determines and implements appropriate interventions.

Respiratory					
**Critical Element:**		Evaluation			Comments
		I	S	R	
1.	**General Assessment**				
	Describes general rationale for accurate vital signs.				
	Obtains complete respiratory assessment:<br>Respiratory rate<br>O_2 saturation<br>Work of breathing: (retractions, head bobbing, nasal flaring, breath sounds, symmetrical chest expansion)				
	Documents accurately the findings of a thorough respiratory assessment in patient record.				
	Identifies trends and changes in the patient's condition that are different from the previous respiratory assessment.				
	Identifies ABNORMAL findings on exam and reports them to preceptor and appropriate physicians.				
2.	**Oxygen Saturation**				
	Demonstrates correct application of oximetry probe and identification of a good wave form.				
	Defines normal limits for oxygen saturation.				
3.	**Administration of Supplemental Oxygen**				
	Identifies respiratory support modality appropriate for patient's level of respiratory distress.				
	Documents level of support in patient medical record.				
	Performs a respiratory reassessment on patient following intervention (application of oxygen modality).				
4.	**Pulmonary Toileting**				
	Performs repositioning and suctioning when necessary.				
	If suctioning is required, selects adequate route and size suction catheter.				

Cardiac Assessment					
**Critical Element:**		Evaluation			Comments
		I	S	R	
5	**General Assessment**				
	Describes general rationale for accurate vital signs.				
	Obtains complete cardiac assessment in addition to heart rate if necessary (ill child): capillary refill, skin color, edema<br>-On one patient is able to identify and record capillary refill				

CLINICAL COMPETENCIES

	Documents accurately the findings of a thorough cardiovascular assessment in patient record.				
	Identifies trends and changes in the patient's condition that are different from the previous cardiac assessment.				
	Identifies ABNORMAL findings on exam and reports them to preceptor and appropriate physicians.				
6	**Heart Rate**				
	Demonstrates use of stethoscope to assess heart rate and physiologic locations to assess a patient's pulse.				
	Defines normal limits for heart rate, and verifies that mechanical tools are accurate.				
	Identifies need for supplemental support if a patient is bradycardic or tachycardic.				
	Identifies support modality appropriate for patient's level of perfusion derangement. Performs positioning, intravenous (IV) placement, fluid resuscitation as indicated.				
	Performs reassessment on patient following intervention.				

MENTAL STATUS					
**Critical Element:**		Evaluation			Comments
		I	S	R	
7	**General Assessment**				
	Describes general rationale for accurate vital signs.				
	Completes a mental status assessment using AVPU (alert, verbal, pain, unresponsive) scoring				
	Accurately documents the findings of a thorough mental status assessment				
	Identifies trends and changes in the patient's condition that are different from the previous respiratory assessment.				
	Identifies ABNORMAL findings on exam and reports them to preceptor and appropriate physicians.				
8	**AVPU**				
	Demonstrates ability to categorize a patient using the AVPU tool.				
	Identifies derangements or changes in mental status and documents this in the medical record.				
	Identifies need for additional support for acute changes in mental status.				
9	**Interventions**				
	Identifies possible causes and interventions appropriate for patient's level of alertness.				
	Documents level of support in patient medical record.				

Partners In Health Inshuti Mu Buzima

CLINICAL COMPETENCIES

	Performs a reassessment on patient following intervention.				

**Temperature**					
**Critical Element:**		Evaluation			Comments
		I	S	R	
1	**General Assessment**				
	Describes general rationale for accurate vital signs.				
	Accurately uses the thermometer.				
	Accurately documents the findings (temperature).				
	Identifies trends and changes in the patient's condition that are different from the previous readings.				
	Identifies ABNORMAL readings on examination and reports them to preceptor and appropriate physicians.				
1	**Interventions**				
	Identifies possible causes and interventions appropriate for a fever or hypothermia.				
	Documents level of support in patient medical record.				
	Rechecks temperature of patient following intervention.				

**Pediatric Early Warning Score (PEWS)**					
**Critical Element:**		Evaluation			Comments
		I	S	R	
1	**General Assessment**				
	Describes general rationale for accurate vital signs.				
	Calculates a PEWS scores.				
	Accurately documents the score.				
	Identifies ABNORMAL scores and reports them to preceptor and appropriate physicians.				
	Accurately identifies and describes the meaning of changes in PEW scores.				
2	**Interventions**				
	Identifies possible causes and interventions.				
	Documents level of support in patient medical record.				
	Performs a reassessment on patient following intervention within the appropriate time from (for PEW score).				

Method of Evaluation: Direct Observation * Comments – if needed please write on back of page

Overall Clinical Evaluation: ___________________________________

Method of Evaluation: Direct Observation * Comments – if needed please write on back of page

Partners In Health Inshuti Mu Buzima

PARTNERS IN HEALTH NURSING • COMPASSION • ADVOCACY • LEADERSHIP

CLINICAL COMPETENCIES

I: Independent S: With Supervision R: Needs Remediation

I, the undersigned, have demonstrated the necessary knowledge, skills, attitudes, values, and/or abilities to be deemed competent in caring for a neonate during admission to the Neonatal Intensive Care Unit. I acknowledge that ongoing development and maintenance of competency is my responsibility and will be evidenced in my Professional Practice Portfolio.

Nurse Signature ....Date...../...../........

Assessor (Preceptor or CNE/F) Name.................................. Signature .... Date...../...../.........
